# Visual Surround Suppression in Schizophrenia

**DOI:** 10.3389/fpsyg.2013.00088

**Published:** 2013-02-28

**Authors:** Marc S. Tibber, Elaine J. Anderson, Tracy Bobin, Elena Antonova, Alice Seabright, Bernice Wright, Patricia Carlin, Sukhwinder S. Shergill, Steven C. Dakin

**Affiliations:** ^1^Institute of Ophthalmology, University College London London, UK; ^2^NIHR Biomedical Research Centre at Moorfields Eye Hospital London, UK; ^3^Institute of Cognitive Neuroscience, University College London London, UK; ^4^Institute of Psychiatry, King’s College London London, UK; ^5^Department of Cognitive, Perceptual and Brain Sciences, University College London London, UK

**Keywords:** schizophrenia, surround suppression, cortex, contrast, luminance, size, orientation, perception

## Abstract

Compared to unaffected observers patients with schizophrenia (SZ) show characteristic differences in visual perception, including a reduced susceptibility to the influence of context on judgments of contrast – a manifestation of weaker *surround* suppression (SS). To examine the generality of this phenomenon we measured the ability of 24 individuals with SZ to judge the luminance, contrast, orientation, and size of targets embedded in contextual surrounds that would typically influence the target’s appearance. Individuals with SZ demonstrated weaker SS compared to matched controls for stimuli defined by contrast or size, but not for those defined by luminance or orientation. As perceived luminance is thought to be regulated at the earliest stages of visual processing our findings are consistent with a suppression deficit that is predominantly cortical in origin. In addition, we propose that preserved *orientation* SS in SZ may reflect the sparing of broadly tuned mechanisms of suppression. We attempt to reconcile these data with findings from previous studies.

## Introduction

Schizophrenia (SZ) is a mental disorder characterized by a range of cognitive, affective, and perceptual symptoms, the diversity of which presents a considerable challenge to any single pathophysiological model of the condition (Cohen and Servan-Schreiber, 1992; Barch and Ceaser, 2012). However, recent studies indicate that visual deficits in SZ may result from abnormalities in *gain control* (Butler et al., 2008), the inhibitory processes by which neurons regulate their overall levels of activity to optimize information transmission (Heeger, 1992). That gain control is an example of a *canonical* neural computation (Carandini and Heeger, 2012), i.e. one that is likely to be repeated across different brain regions and modalities, makes it a potential candidate for involvement in the wide range of symptoms that characterize SZ.

**Figure 1:**
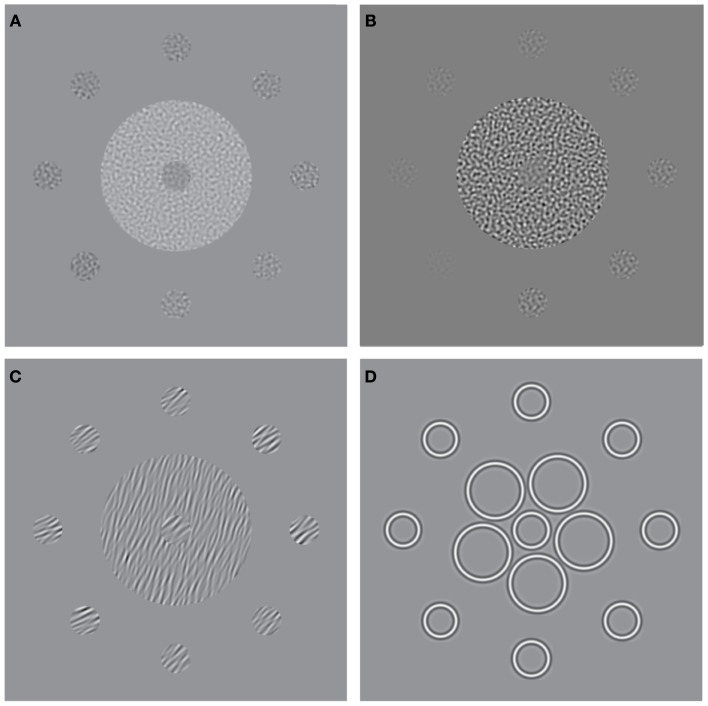
**Stimuli used to measure surround suppression for judgments of (A) luminance, (B) contrast, (C) orientation, and (D) size**. The stimulus consists of a central patch (the “reference”) presented within a surround of **(A)** higher luminance, **(B)** higher-contrast, **(C)** more anti-clockwise orientation, and **(D)** larger elements. Here, for the purpose of illustration, each stimulus is also surrounded by eight test-patches at different signal levels. A typical perceptual match to the central reference-patch is shown at “12 O’clock” whereas the true/physical match is at “6 O’clock.”

In terms of visual processing, gain control is thought to play a critical role in *contextual effects*, whereby the presence of a surround influences or biases the perception of a target (Albright and Stoner, 2002). There is evidence that such phenomena are reduced or absent in people with SZ (Silverstein et al., 2000). For example, the perceived contrast of a target is normally reduced when embedded in a high contrast surround (Figure 1B) – an instance of a more general phenomenon known as *surround suppression* (SS; Chubb et al., 1989). However, patients with SZ are much less susceptible to this effect. As a result, under conditions that would normally lead to SS, patients select a perceptual match that is closer to veridical than do controls (Dakin et al., 2005; Yoon et al., 2009, 2010; Barch et al., 2012). (See Figure 1 legend).

Converging evidence from psychophysical (Solomon et al., 1993), electrophysiological (Ohtani et al., 2002; Haynes et al., 2003) and functional imaging (Williams et al., 2003; Zenger-Landolt and Heeger, 2003) studies suggest that SS is mediated by the inhibition of a neuron’s response to a stimulus by the pooled activity of cells in surrounding cortex (Heeger, 1992; Solomon et al., 1993; Xing and Heeger, 2001). Abnormal SS in SZ is therefore consistent with reduced levels of cortical inhibition (Butler et al., 2008). This hypothesis is supported by the finding that impoverished contrast SS in SZ correlates with a visuo-cortical deficit of gamma-aminobutyric acid (GABA; Yoon et al., 2010), the brain’s primary inhibitory neurotransmitter (see Wassef et al., 2003 for a review of GABAergic models of SZ). Further, we have previously suggested that abnormal performance on a number of visual tasks, e.g. contour integration and visual crowding, can be explained by reduced levels of cortical suppression from a stimulus’ surround (Robol et al., in press).

Analogous SS effects, which may involve similar mechanisms of gain control, have also been demonstrated for visual dimensions other than contrast (Figures 1A,C,D). For example, the brightness of a target is reduced when it is embedded in a high luminance surround (Figure 1A; Adelson, 1993), the perceived orientation of a target is shifted when it is presented within a surround with a different orientation (Figure 1C; Wenderoth and Johnstone, 1988), and the perceived size of a circle is reduced by the presence of large flanking circles (Figure 1D; the Ebbinghaus illusion; Weintraub, 1979). Although the extent to which these effects rely on common mechanisms is not well understood (Webb et al., 2005; Smith, 2006), there is evidence for multiple gain control processes operating at different levels within the visual stream. Whilst luminance gain control is largely mediated by retinal processes (Shapley and Enroth-Cugell, 1984), the locus of SS for judgments of size, orientation and motion is thought to reside further downstream in striate and extra-striate areas once signals from the two eyes have converged. Thus, SS effects for these latter dimensions survive dichoptic presentation, i.e. when the target and surround are presented separately to different eyes (Marshak and Sekuler, 1979; Mather and Moulden, 1980; Wade, 1980; Song et al., 2011). This is not the case for contrast SS however, which only persists if the target and surround are presented to the same eye, implicating an intermediary locus of contrast gain control in pre-cortical or early cortical areas (Chubb et al., 1989).

There is evidence to suggest that several of these SS effects may be diminished in SZ, potentially implicating a widespread deficit in gain control mechanisms. Thus, in addition to well-documented abnormalities in contrast SS as described above (Dakin et al., 2005; Yoon et al., 2009, 2010; Barch et al., 2012), reduced SS is seen for judgments of motion direction in patients with SZ (Tadin et al., 2006) as well as judgments of size in patients with disorganized SZ (Uhlhaas et al., 2006a,b) and non-clinical adults who score highly on a disordered thought sub-score of the Schizotypal Personality Questionnaire (Uhlhaas et al., 2004). However, not all findings reported are consistent with the notion of a widespread deficit. One study attributed *elevated* motion SS effects to individuals with SZ (relative to controls; Chen et al., 2008), and in a recent report of SS effects in the luminance, size, contrast, orientation, and motion domains, weakened contextual modulations in SZ were only reported for judgments of contrast (Yang et al., 2013). Hence, there is conflicting evidence for a general versus a dimension-specific deficit in contextual modulation in SZ.

One problem in establishing the generality of SS deficits in SZ, is that with the exception of a single report (Yang et al., 2013), data for the various versions of the task have typically been obtained using different patient groups, sample sizes, and/or experimental paradigms, thereby hindering direct comparison. To address this limitation, we assessed SS in a single patient group (*n* = 24) using a standardized battery of tasks (Figures 1A–D) and stimuli designed to probe visual function at multiple stages of the processing hierarchy. Observers made judgments of relative luminance, contrast, orientation and size in the context of a reference stimulus embedded in a suppressive surround. We hypothesized that reduced SS stems from a generalized deficit in SZ, and consequently, predicted that patients would be less influenced than controls by the presence of a surround for all four judgment types.

## Materials and Methods

### Observers

Twenty-four observers with SZ (eight female; mean age = 39.96 ± 9 years; mean I.Q. = 102 ± 10) and 24 age-/sex- and IQ-matched controls (mean age = 38.21 ± 12 years; mean I.Q. = 107 ± 9) gave informed written consent to take part in this study (see Table 1). Patient and control groups did not differ significantly with respect to age [*t*(46) = −0.58; *P* = 0.57] or I.Q. [*t*(46) = 1.74; *P* = 0.09]. Patients were recruited from inpatients at the Churchill London Clinic (*n* = 5) and from outpatients at the Institute of Psychiatry (IoP); all had been diagnosed with SZ according to DSM-IV criteria. At the IoP and Churchill London Clinic clinical assessments were undertaken by a Masters level research nurse and clinical psychologist, respectively, both of whom have extensive knowledge and training in the field. Of the 24 patients tested, 12 were diagnosed with paranoid SZ; however, none of the other patients fell firmly into any other specific sub-category. Details of patients’ medication are given in Table 1. Ethics approval was granted by the UK National Research Ethics Committee.

**Table 1:** **Patient details, including medication type (Med), medication dose (Dose; chlorpromazine equivalent in mg/day), diagnosis (SZ, schizophrenia; PS, paranoid schizophrenia), intelligence quotient (I.Q.), total scores on tPANSS, scores for the positive symptoms of the PANSS test (tPSS), scores for the negative symptoms of the PANSS test (tNSS), scores for the general symptoms of the PANSS test (tGSS), scores on a cognitive factor which overlaps heavily with the concept of disorganization syndrome (tDIS) and scores for item P2 on the PANSS test, “conceptual disorganization” (DIS)**.

Sex	Age	Med	Dose	Diag	I.Q.	tPANSS	tPSS	tNSS	tGSS	tDIS	DIS
M	55	Typ	200	PS	110	76	21	17	38	11	4
M	48	Atyp	300	PS	84	74	29	11	34	10	2
M	32	Atyp	500	PS	90	75	19	25	31	9	3
M	23	Typ	1000	PS	99	85	21	22	42	9	2
M	28	Atyp	600	PS	105	76	15	26	35	10	4
M	28	Atyp	1000	SZ	106	58	12	20	26	9	1
F	44	Atyp	400	SZ	103	58	10	16	32	13	2
M	28	Atyp	200	SZ	101	40	7	15	18	8	1
F	37	Atyp	800	SZ	100	100	20	28	52	15	4
M	46	Atyp	400	PS	100	67	18	21	28	10	1
F	30	Atyp	400	SZ	111	57	14	18	25	10	2
F	49	Typ	750	PS	112	61	20	12	29	12	2
M	51	Atyp	1000	PS	89	40	12	9	19	8	2
M	34	Atyp	200	SZ	111	42	7	14	21	8	1
M	34	Atyp	200	PS	111	48	8	14	26	8	1
M	44	Atyp	600	PS	117	47	8	12	27	8	1
M	51	Atyp	150	SZ	95	73	16	25	32	11	1
M	29	Atyp	800	SZ	100	63	13	18	32	10	1
M	43	Typ	133	SZ	101	45	8	17	20	6	1
F	44	–	0	SZ	112	74	20	13	41	15	1
F	41	Atyp	1400	PS	117	55	12	17	26	11	2
F	46	Atyp	133	PS	94	70	21	12	37	14	3
M	53	Atyp	200	SZ	111	46	7	18	21	10	1
F	41	Atyp	250	SZ	81	32	7	9	16	7	1
Mean	39.96	–	484	–	102.5	60.92	14.38	17.04	29.5	10.08	1.83
std	9.32	–	363.6	–	9.94	16.61	6.1	5.36	8.65	2.38	1.05

*Only one patient had a history of substance abuse. Typ, typical; Atyp, atypical; diag, diagnosis; std, standard deviation*.

### Apparatus

Stimuli were presented on a CRT monitor (LaCie Electron Blue 22), which was viewed at 120 cm at a spatial and temporal frequency of 1024 × 768 pixels and 75 Hz, respectively. The monitor was fitted with a Bits++ box (Cambridge Research Systems) operating in Mono++ mode to give true 14-bit contrast accuracy. The display was calibrated with a Minolta LS110 photometer and linearized using custom software using a look-up table. Experiments were run in the Matlab programming environment (MathWorks, Cambridge, MA, USA) – in conjunction with Psychtoolbox (Brainard, 1997; Pelli, 1997) – on an Apple MacBook Pro computer.

### Procedure

All experiments (presented in pseudo-random order) involved a two-(temporal)-interval-forced choice (2-IFC) task in which the observer had to report which of two stimuli (the reference or target) was “the brighter” (luminance task), “stronger” (contrast task), “tilted closer to the horizontal” (orientation task), or “larger” (size task). Observers gave a verbal response on each trial, which the experimenter then relayed to the computer by button-press. The entire testing session (including obtaining of consent and debriefing) lasted approximately 1.5 h, with around 20 min allocated for each experiment. For examples of the stimuli used see Figure 1, but note that in the actual experiment the reference and target were presented centrally in two separate temporal intervals. Stimulus presentation time was fixed at 500 ms with an inter-stimulus interval of 1250 ms. Fixation was assisted by the presence of a central black cross, which turned white when stimuli were presented onscreen.

The target stimulus consisted of an isolated disk with a radius subtending 0.34° of visual angle. The reference was a disk of identical dimensions embedded in an annular surround with an outer radius of 1.91°. For all four experiments the reference (surrounded) stimulus did not vary across trials, whereas the test stimulus varied either in luminance, contrast, orientation, or size, depending on experiment. The test value presented on any single trial was controlled by an adaptive method (Watt and Andrews, 1981) that sought to probe responses that would be maximally informative about the slope and offset (bias) of the underlying psychometric function, which was approximated by a cumulative Gaussian (see below). All runs consisted of 64 trials, during which the signal level (luminance, contrast, orientation, or size of the test) was manipulated.

**Figure 2:**
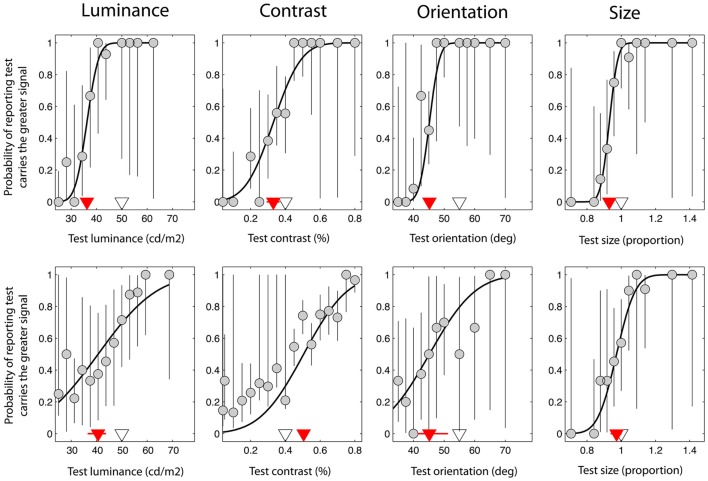
**Data taken from a single control observer (upper plots) and a single observer with schizophrenia (lower plots)**. These were selected on the basis that they were typical of group trends. Red triangles denote the point of subjective equality (PSE) on the abscissa, which represents the test signal level at which the test and reference were perceptually matched. The white triangles denote the test signal when test and reference were physically (i.e. veridically) matched. Error bars represent 95% confidence intervals for parameter estimates obtained through boot-strapping of the observer’s responses.

For each task and each individual the probability that the observer reported that the test had a higher signal than the reference (Figure 2 upper left panel) was plotted against the signal level of the test (luminance in the luminance task for example). Data follow a sigmoidal distribution that is well fit by a cumulative Gaussian function defined by two parameters: a slope and a bias. The form of the cumulative Gaussian captures the fact that when the test is considerably brighter than the reference, observers (almost) always report the test as having the higher signal, whereas when the reference is much brighter than the test, participants (almost) never report the test as having the higher signal. The slope of the line connecting these two asymptotes defines how sensitive the observer is to changes in the relative signal of the test and reference. A steep slope represents high sensitivity, such that small changes in the signal difference elicit large changes in the responses of the observer. Sensory *threshold* is the inverse of the slope of a cumulative Gaussian function, and defines the difference in test signal and reference-signal that is needed for the observer to respond correctly on a certain percentage of trials (defined here as 84%). For example, a threshold of 5 cd/m^2^ implies that the test and reference must differ in luminance by 5 cd/m^2^ for the observer to correctly discriminate their brightness on 84% of trials. Thus, a high threshold represents poor performance.

The second parameter of the cumulative Gaussian, the offset or bias, defines the function’s mid-point. Also known as the point of subjective equality (PSE), it is the test signal level at which the observer reports the test as having the higher signal on 50% of trials, i.e. the point at which the test and reference are indistinguishable for the dimension of interest, and hence are perceived as matched. For the experiments reported here, the bias is expressed relative to the *veridical* match point and represents the shift in the perceived signal level of the reference as a result of being embedded in the surround. A negative bias is therefore indicative of a strong suppressive effect. For example, a bias of −10 cd/m^2^ would imply that the perceived luminance of the reference is reduced by 10 cd/m^2^ when embedded in the surround.

Ninety-five percent confidence intervals (CIs) and the standard deviation of fit parameter estimates (threshold and bias) were obtained through boot-strapping (re-sampling) and re-fitting of the raw data. These were subsequently *Z*-transformed (expressed as units of standard deviation relative to the mean) and used to derive weightings for each parameter estimate; these followed an inverse cumulative Gaussian such that parameter estimates associated with smaller CIs (i.e. higher confidence) contributed most heavily to the analyses [weighted *t*-tests and correlations were undertaken using Matlab and SPSS statistical analysis software (version 18.0; SPSS Inc., Chicago, IL, USA)]. In addition, data with an associated confidence interval for the bias parameter >2.58 standard deviations from the mean were excluded on the basis that they were extreme outliers and reflected data that were poorly fit by the cumulative Gaussian model. (The mean ± 2.58 standard deviations captures the 1st to 99th percentile of a normal probability distribution function). All statistical analyses undertaken employed two-tailed tests, unless stated otherwise.

### Stimuli

For the **luminance task**, the reference patch, the test patch, and the surround-annulus were random-noise filtered with a spatial-frequency (SF) band-pass LogGabor filter passing a mean SF of 11.25 c/deg. with a bandwidth of 0.4 octave (the σ of the log-Gaussian defining the filter in the fourier domain). Michelson contrast was fixed at 50%. The mean-luminance of the reference and its uniform background were fixed at 50 cd/m^2^ and the reference-surround at 75 cd/m^2^. The luminance of the test fell between 37.5 and 62.5 cd/m^2^ depending on performance.

For the **contrast task**, the reference, test, and surround again consisted of LogGabor filtered noise (same characteristics as in the luminance task, except that luminance was now fixed at 50 cd/m^2^ and the contrast of the reference and surround were set at 40 and 95%, respectively). The contrast of the test patch varied between 4 and 80%.

For the **orientation task**, the reference, test, and surround were comprised of similar-(LogGabor) filtered noise which had also been orientation-limited using a wrapped Gaussian weighted pass-band with a 5° (σ) bandwidth. Test luminance was 50 cd/m^2^ and contrast 95%. The reference had a mean orientation of 55° (anti-clockwise from horizontal), and was embedded in a 75° surround. The test orientation varied between 35° and 75°.

For the **size task** the reference was a ring with a radius of 0.34° surrounded by four large circles, each with a radius of 0.7° with a 1.1° separation. The test ring radius varied within a range of 0.22°–0.44°. Ring edges had a sinusoidal profile with a SF of 11.25 c/deg. presented at 95% contrast and an average luminance of 50 cd/m^2^.

## Results

### Individual data

Figure 2 shows data from one control observer (top row) and one observer with SZ (bottom row). On the abscissa the test signal level (i.e. luminance, contrast, orientation, or size of the test) is plotted against the probability that the observer reported that the test was brighter, higher-contrast, clockwise, or larger compared to the reference (respectively). Data have been fit with a cumulative Gaussian function (solid black line) defined by two parameters: the threshold and bias (the positioning of the curve along the abscissa). In addition, 95% CIs were generated for each of these parameters using a method of boot-strapping. The threshold indicates the smallest difference in signal between the reference and the test that would allow the observer to correctly discriminate the two on 84% of trials. The bias (red triangle) (with associated 95% CIs; red lines) represents the test signal level for which the reference and test are *perceived* as matched, so that the observer reports the test as brighter on 50% of the trials. The *actual* signal level of the reference is denoted by a white triangle for comparison; if judgments were unbiased, the red and white triangles would coincide. For the luminance task (Figure 2; upper left plot), note that the perceived match point (red triangle) lies to the left of the actual reference stimulus level (white triangle). Thus, for the test to be perceptually matched to a reference with a luminance of 50 cd/m^2^, it must have a luminance of approximately 36 cd/m^2^, consistent with the mid-gray reference patch appearing darker when presented in a bright surround.

### Confidence of parameter estimation

Before comparing thresholds and biases between the control and patient groups, data were filtered to remove any values that were associated with high uncertainty. Thus, parameter estimates with an associated CI that was >2.58 standard deviations from the group mean were excluded from the analysis (see Materials and Methods). This resulted in the exclusion of 7% of the patients’ data, compared to only 1% of the control group’s data. Following exclusion of these extreme outliers, control, and patient CIs were compared across the four tasks using a multivariate analysis of variance (MANOVA) with four dependent variables (performance on the luminance, contrast, orientation, and size tasks) and one independent variable (group at two levels: patients and controls). Bias and threshold CIs were analyzed independently. These revealed a main effect of group for biases [*F*_(4,33)_ = 2.92; Wilk’s λ = 0.74, *P* = 0.04, partial η^2^ = 0.26], but not for thresholds [*F*_(4,33)_ = 2.5; Wilk’s λ = 0.77, *P* = 0.06, partial η^2^ = 0.23]. To explore these findings further, a series of independent-samples *t*-tests were undertaken. Significance was defined at an alpha level of 0.0125 (correction for four multiple comparisons). CIs for both the bias and threshold parameter were found to be significantly elevated in the patients (relative to controls) for judgments of orientation only (*P*s < 0.01; Table 2). Consequently, after filtering for extreme outliers, we find some evidence for a poorer fit to the patient group data, although the effect is not consistent across tasks.

**Table 2:** **Independent-samples *t*-tests comparing patient and control group 95% confidence intervals for biases and thresholds**.

Parameter	Task	*t*	*df*	*P*	*d*
Bias CIs	Luminance	2.6	40	0.013	0.61
	Contrast	0.51	44	0.61	0.14
	Orientation	2.9	40	<0.01*	0.63
	Size	1.91	45	0.06	0.48
Threshold CIs	Luminance	2.46	40	0.02	0.56
	Contrast	1	44	0.33	0.24
	Orientation	2.9	40	<0.01*	0.65
	Size	2.43	45	0.02	0.58

Alpha level = 0.0125, reflecting correction for four multiple comparisons. *P*, significance level; *d*, Cohen’s *d*; *significant effect for given alpha level.

### Group biases and thresholds

**Figure 3:**
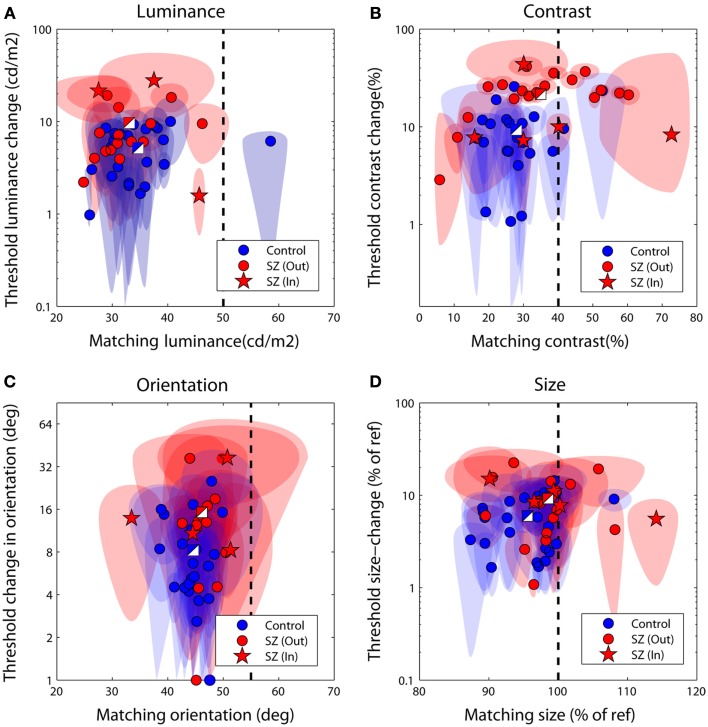
**Discrimination performance for the four judgments of relative (A) luminance, (B) contrast, (C) orientation, and (D) size**. Each data-point plots bias (the test level leading the isolated test to be perceptually matched to the surrounded reference) against threshold (the difference in reference and test signal required to successfully discriminate the two on 83% of trials). The performance of control observers is plotted in blue, and of patients in red; two-toned squares show group averages. Ellipses denote 95% confidence intervals for parameter estimates. The vertical dotted line denotes a veridical (unbiased) match. Large negative biases reflect strong suppressive effects of the surround whilst larger threshold values (on the ordinate axis) reflect poorer performance. In, inpatients; Out, outpatients.

Figures 3A–D plot bias against threshold data derived from the psychometric functions fit individually to each observer’s data. Blue/white and red/white square data-points represent group averages for the control and patient groups respectively. Notice that for all tasks the patient group data fall above – and with the exception of the luminance task – to the right of the control group data. To explore this separation quantitatively we used MANOVA with four dependent variables (performance on the luminance, contrast, orientation, and size tasks) and one independent variable (group at two levels: patients and controls). Biases and thresholds were analyzed separately. Analyses highlighted a significant main effect of group on biases [*F*_(4,33)_ = 2.74; Wilk’s λ = 0.75, *P* = 0.05, partial η^2^ = 0.25] as well as thresholds [*F*_(4,33)_ = 2.96; Wilk’s λ = 0.74, *P* = 0.03, partial η^2^ = 0.26].

To examine which tasks underlie these effects a series of independent-samples *t*-tests (weighted by parameter confidence) were carried out to compare group biases and group thresholds on individual tasks. Statistical significance was defined at an alpha level of 0.025, reflecting adjustment of the standard value (0.05) for a single-tailed test and Bonferroni correction for four multiple comparisons (reflecting a total of four separate tasks, with biases and thresholds once again tested independently). Single-tailed tests were employed since on the basis of previous literature our hypothesis was unidirectional: with the exception of a single study of motion (which was not tested here; Chen et al., 2008), measures of SS have only ever highlighted significant associations between *high* schizotypal traits (or SZ itself) and *reduced* contextual modulation (Uhlhaas et al., 2004, 2006a,b; Dakin et al., 2005; Tadin et al., 2006; Yoon et al., 2009, 2010; Barch et al., 2012; Yang et al., 2013). Thresholds were found to be significantly higher in patients with SZ (relative to controls) for the contrast judgments only (*P* < 0.001; see Table 3 and Figure 3B). For biases there was a general trend for all data to fall to the left of the zero bias line (veridical match), reflecting the effect of a suppressive surround. However, relative to control values, these biases were found to be significantly reduced (less negative) in the patients with SZ for both contrast and size judgments (*P* = 0.025 and *P* = 0.02, respectively). This suggests that the patients with SZ were less susceptible to the suppressive effects of context in both the contrast and size domains.

**Table 3:** **Weighted *t*-tests comparing patient and control group biases and thresholds**.

Parameter	Task	*t*	*df*	*P*	*d*
Threshold	Luminance	2.105	40	0.04	0.48
	Contrast	5.150	44	<0.001*	0.99
	Orientation	1.021	40	0.31	0.27
	Size	1.421	45	0.16	0.31
Bias	Luminance	0.98	40	0.33	0.2
	Contrast	2.3	44	0.025*	0.68
	Orientation	1.59	40	0.12	0.39
	Size	2.42	45	0.02*	0.52

Alpha level = 0.025, reflecting correction for single-tailed test and four multiple comparisons. *P*, significance level; *d*, Cohen’s *d* with individual values weighted by parameter confidence; *significant effect for given alpha level.

**Figure 4:**
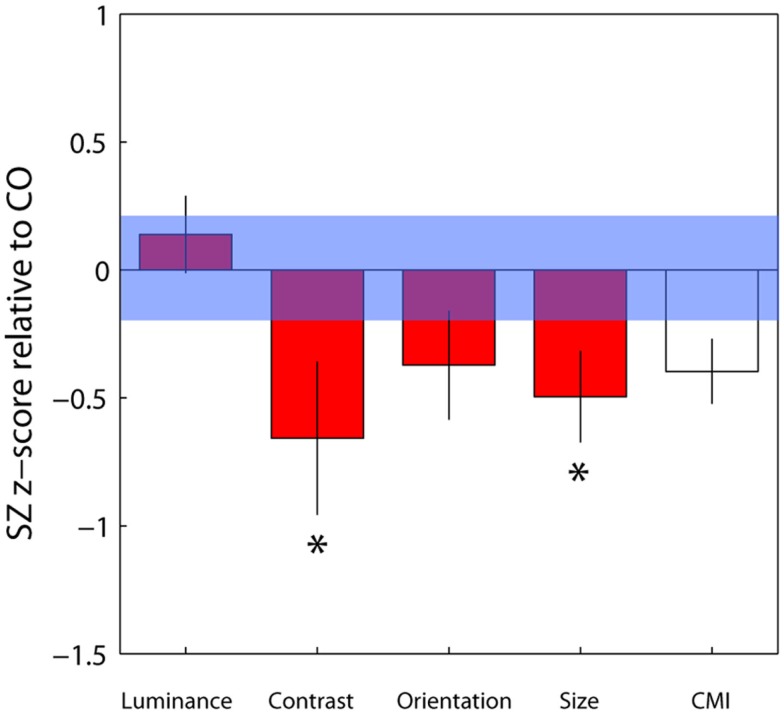
**Bias data from the schizophrenia group have been converted into *z*-scores relative to control group means and standard deviations for each of the four tasks, with individual parameter estimates weighted by their associated confidence interval**. Negative and positive values, respectively, denote weaker and stronger contextual effects in the patient group. For each patient, a mean of these four standardized *z*-scores was calculated, generating a contextual modulation index (CMI) that represents a measure of *general* susceptibility to surround suppression (white bar). Error bars indicate the standard error of the mean (SEM) of the patient group; the blue shaded region indicates the SEM of the control group.*Significant effect at the 5% level following correction for multiple comparisons and single-tailed tests.

To compare contextual modulation effects across tasks and to facilitate comparison of effect sizes with previous studies, SZ group biases were re-plotted following *z*-score transformation relative to control group data, as in Yang et al. (2013), but with all parameters weighted by confidence (Figure 4). Note that this is a signed measure of the effect size: a variation of Cohen’s *d*, in which group differences are normalized by the control group variance as opposed to the pooled variance (also known as Glass’s delta). Negative values reflect reduced suppression in the patients relative to controls. Reinforcing the findings of statistical comparisons, the data show reduced suppression in the contrast, orientation, and size domains, with the largest effects for judgments of relative contrast and size (0.68 and 0.52, respectively; Figure 4 and Table 3). Following Yang et al. (2013), we also generated a *general* contextual modulation index (CMI) for each patient; this was calculated by averaging individuals’ *Z*-transformed biases across all four tasks. Once again, negative values imply a suppressive effect of the surround. The group mean CMI for the patient group was −0.4 with a standard deviation of 0.62 (Figure 4). A comparison of control and patient CMIs reveal a significant difference at the single-tailed, but not two-tailed, level [*t*_(46)_ = 1.71, *P* = 0.09].

### Correlations between tasks

If SS along distinct visual dimensions relies on shared mechanisms of gain control one might expect to find correlations between biases from distinct SS tasks. In Table 4, weighted Pearson’s correlation coefficients and associated *P* values are presented for correlations between biases for all four tasks. We also tested for correlations between thresholds and biases in order to check that the reduced biases we report in the patient group are not simply an artifact of elevated thresholds. If this were the case we would expect biases and thresholds to correlate within each SS task. Statistical significance was defined at an alpha level of 0.0063, reflecting Bonferroni correction for eight multiple comparisons/parameter. Analyses show that none of the biases from one task correlated with biases from another task (all *P*s > 0.08), a finding that is consistent with independent mechanisms of SS across the different dimensions. In addition, we report no significant correlations between thresholds and biases on any single task (all *P*s > 0.16), suggesting any group differences in biases are unlikely to be driven by an artifact of elevated thresholds. Interestingly, there was a strong and significant correlation between luminance thresholds and contrast thresholds (*P* = 0.005).

**Table 4:** **Inter-correlations between biases and thresholds for each of the four tasks tested**.

		1	2	3	4	5	6	7	8
1. Luminance bias	R	–							
	P	–						
2. Contrast bias	R	−0.07	–						
	P	0.67	–					
3. Orientation bias	R	−0.06	−0.1	–					
	P	0.71	0.52	–				
4. Size bias	R	−0.16	−0.01	−0.28	–				
	P	0.3	0.94	0.08	–			
5. Luminance thresh	R	0.07	−0.26	−0.26	0.24	–			
	P	0.66	0.11	0.11	0.14	–		
6. Contrast thresh	R	0.4	−0.2	−0.1	0.19	0.43	–		
	P	0.009	0.16	0.52	0.21	0.005*	–	
7. Orientation thresh	R	0.02	−0.11	0.14	0.06	0.32	0.25	–	
	P	0.93	0.51	0.39	0.71	0.05	0.11	–
8. Size thresh	R	0.15	−0.06	−0.07	0.2	0.24	0.2	0.38	–
	P	0.34	0.7	0.65	0.19	0.14	0.18	0.01	–

Alpha level = 0.0063, reflecting correction for 8 multiple comparisons/task. *Significant effect for given alpha level.

### Correlations between task performance, clinical symptoms and medication dose

To determine whether group differences in bias and threshold could be attributed to the patients’ anti-psychotic medication we also tested for correlations between patients’ daily medication dose (converted into chlorpromazine equivalents; Woods, 2003; Taylor et al., 2012) and scores on behavioral measures including CMIs (Table 5). Statistical significance was defined at an alpha of 0.025, reflecting adjustment of the standard value (0.05) for a single-tailed test and Bonferroni adjustment for clinical symptoms multiple comparisons. Single-tailed tests were employed since our experimental hypothesis – that *elevated* SS in the patients can be attributed to medication – was unidirectional. Weighted Pearson’s correlation coefficients and associated *P* values indicate that none of the comparisons approached statistical significance (all *P*s > 0.25). Consequently, we find no evidence that differences between patient and control group data can be attributed to patients’ medication.

**Table 5:** **Correlations between medication dose (chlorpromazine equivalent in mg/day) and individual behavioral measures (biases and thresholds)**.

		LumB	ContB	OrientB	SizeB	LumT	ContT	OrientT	SizeT	CMI
Dose	R	−0.16	−0.07	−0.25	−0.14	−0.26	−0.08	−0.29	0.17	−0.04
	P	0.52	0.73	0.31	0.53	0.3	0.71	0.25	0.45	0.87

*Alpha level = 0.025, reflecting correction for single-tailed test and four multiple comparisons. Lum, luminance; Cont, contrast; Orient, orientation; B, bias; T, threshold; CMI, contextual modulation index*.

Finally, to determine whether any of the behavioral measures tested correlated with symptom severity we calculated weighted Pearson’s correlation coefficients between behavioral measures (including CMIs) and individual total PANSS scores the positive and negative symptoms scale (tPANSS), as well as negative, positive, general psychopathology sub-scale scores, and a cognitive factor that overlaps heavily with the concept of disorganization syndrome (Lindenmayer et al., 1994). This cognitive factor is based on the scoring of patients to a subset of questions in the PANSS test [Poor attention (G11); mannerisms and posturing (G5); conceptual disorganization (P2); difficulty in abstract thinking (N5); disorientation (G10)] and has previously been shown to correlate with poor performance in SZ on a contour integration task (Silverstein et al., 2000). In addition, following Uhlhaas et al. (2006a), we also looked for correlations between task performance and scores on question P2 of the PANSS test (conceptual disorganization; DIS). The alpha level was set to 0.0083, reflecting Bonferroni correction for six multiple comparisons (a total of six PANSS measures recorded). No significant correlations were found between behavioral measures and any of the PANSS scores (Table 6).

**Table 6:** **Correlations between the behavioral measures (biases and thresholds) and PANSS scores (patient data only)**.

		tPANSS	tPSS	tNSS	tGSS	tDIS	DIS
LumB	R	0.12	0.09	0.1	−0.06	−0.33	0.36
	P	0.64	0.73	0.7	0.81	0.19	0.14
ContB	R	0.16	−0.06	0.22	0.18	0.04	−0.15
	P	0.48	0.78	0.31	0.41	0.85	0.5
OrientB	R	−0.14	−0.32	−0.02	−0.21	−0.14	−0.39
	P	0.57	0.2	0.93	0.41	0.59	0.11
SizeB	R	−0.09	0.03	−0.01	−0.1	0	−0.03
	P	0.67	0.88	0.96	0.67	0.99	0.89
LumT	R	0.09	0.07	−0.13	0.06	−0.01	−0.15
	P	0.71	0.78	0.6	0.81	0.97	0.56
ContT	R	−0.04	−0.07	−0.15	0.19	0.41	−0.16
	P	0.86	0.74	0.49	0.39	0.05	0.47
OrientT	R	−0.08	−0.23	0.02	−0.08	−0.24	−0.16
	P	0.75	0.35	0.93	0.75	0.34	0.52
SizeT	R	−0.21	−0.07	−0.45	−0.19	−0.03	0.07
	P	0.34	0.76	0.03	0.39	0.9	0.72
CMI	R	−0.05	−0.17	0.18	−0.11	−0.24	−0.05
	P	0.8	0.42	0.4	0.6	0.25	0.83

*Alpha level = 0.0083, reflecting correction for six multiple comparisons. R, Pearson’s correlation coefficient; *P*, significance level; Lum, luminance; Cont, contrast; Orient, orientation; B, bias; T, threshold; CMI, contextual modulation index*.

## Discussion

We report that patients were more accurate (less biased) than healthy controls for judgments of relative contrast and size, implicating a reduced influence of surrounding context within these visual domains. However, with respect to our stated hypothesis (that attenuated contextual modulation is a general property of the visual system in SZ), we do not report evidence for a deficit across *all* tasks employed; patients showed SS effects that were statistically indistinguishable from controls’ for stimuli defined both by luminance and orientation.

These findings are consistent with a number of studies that have reported comparable deficits in contextual modulation using analogous SS tasks in studies of SZ and schizotypal traits. Thus, reduced SS effects have previously been reported for judgments of relative contrast (Dakin et al., 2005; Yoon et al., 2009, 2010; Barch et al., 2012) and motion (Tadin et al., 2006) in patients with SZ, as well as size in patients with disorganized SZ (Uhlhaas et al., 2006a,b) and non-clinical adults who scored highly on a disordered thought sub-score of the Schizotypal Personality Questionnaire (Uhlhaas et al., 2004). Although this implicates attenuated SS in SZ for a number of visual dimensions, not all studies support this conclusion. Using random-dot motion stimuli Chen et al. (2008) reported a reversed pattern of effects: *elevated* SS in patients with SZ relative to matched controls. In addition, as the majority of these studies have typically employed distinct experimental paradigms and heterogeneous patient groups it is difficult to compare findings across tasks and draw general conclusions.

One recent publication has attempted to address this limitation in the literature directly. A study by Yang et al. (2013) used a similar design and experimental approach to test a single group of patients with SZ on a batch of SS tasks that measured contextual modulations for judgments of relative luminance, contrast, orientation, size *and* motion. They reported attenuated contrast SS in the patient group relative to controls – with a similar effect size to our own: Cohen’s *d* = 0.64 compared to 0.68 – but found no evidence for group differences on any of the other dimensions tested (see points raised below however). This effect size is considerably weaker than that found in the original study by Dakin et al. (2005), but larger than reported by Barch et al.(2012; Cohen’s *d* = 0.31). This is unsurprising however: whilst Barch et al. (2012) only tested stable outpatients and Dakin et al. (2005) forensic inpatients (who were chronically ill), our own data were based on a mixture of inpatient and outpatient populations. Taken together, these findings support the notion that despite preserved mechanisms of luminance gain control in SZ, contrast SS is attenuated relative to controls. Further, whilst several studies have demonstrated an analogous deficit for size SS in a subgroup of patients and non-clinical adults with disordered thought, there is inconsistent and somewhat contradictory evidence as to whether or not orientation and motion judgments are similarly affected (Tadin et al., 2006; Chen et al., 2008; Yoon et al., 2009, 2010; Yang et al., 2013).

One potentially important distinction between Yang et al. (2013) and other studies (including our own), is that with the exception of the motion task, unlimited exposure times were used: stimuli remained onscreen until the observer gave a response, a design that may be suboptimal for uncovering group differences. With respect to orientation at least, the magnitude of SS is dependent on stimulus presentation time (Calvert and Harris, 1988), such that the effect diminishes at durations >100 milliseconds. Consequently, prolonged exposure to the stimulus may reduce the likelihood of uncovering group differences by minimizing baseline biases, thereby risking floor effects. In support of this possibility, whilst Yang et al. (2013) report that a high-signal (oriented) surround shifted the perceived orientation of a target by an average of 2.86° in the control group, we report a mean shift of 10.84° using our briefly presented stimuli. Despite these discrepancies, both the work of Yang et al. (2013) and the findings of our own study point to the existence of preserved mechanisms of luminance gain control in SZ: patients showed normal SS effects for luminance judgments. This raises the possibility that the notional visual dysfunction in SZ may be restricted to cortical as opposed to pre-cortical loci and places a theoretical lower bound on the deficit. Relative to other visual dimensions, e.g. motion, orientation, and size (Marshak and Sekuler, 1979; Mather and Moulden, 1980; Wade, 1980; Song et al., 2011), luminance signals are processed at the very earliest stages of the visual hierarchy, within the retina and lateral geniculate nucleus (LGN; Shapley and Enroth-Cugell, 1984). Indeed, there is some support from post-mortem anatomical studies of SZ for deficits within the visual system being restricted to cortical loci: whilst there is a 25% reduction in neuron number (and 22% reduction in total volume) in the primary visual cortex in patients with SZ (relative to controls; Dorph-Petersen et al., 2007), no such deficit was found in the LGN (Lesch and Bogerts, 1984; Selemon and Begovic, 2007; Dorph-Petersen et al., 2009) – an important pre-cortical site of luminance gain control.

Meta-analyses of magnetic resonance imaging (MRI) voxel-based morphometric studies, which quantify regional differences in gray matter volume, do not highlight such a distinction between cortical and pre-cortical deficits in SZ however (Ellison-Wright et al., 2008; Honea et al., 2008; Fornito et al., 2009). Whilst frontal and temporal abnormalities are strongly associated with SZ (see Hulshoff Pol and Kahn, 2008 for a review), several subcortical – including thalamic-loci have also been implicated (Andreasen et al., 1994; Blennow et al., 1996; Staal et al., 1998), although at least some of these may reflect secondary effects of treatment with anti-psychotic medication (Dazzan et al., 2005). Morphometric (Wright et al., 1995) and diffusion tensor imaging studies (Shergill et al., 2007) have also highlighted a number of white matter (neural fiber) defects in SZ that extend to fronto-thalamic connections (Suzuki et al., 2002). Thus, although reported anatomical abnormalities in SZ are commonly *cortical* in nature, the existing literature clearly does not rule out the possibility of related deficits in subcortical structures.

What is to be made of our finding that levels of *orientation* SS also did not differ significantly between patient and control groups, a finding that corroborates the work of Yang et al. (2013)? This was contrary to our prediction: orientation SS (Figure 1C) is putatively driven by inhibition between cell populations tuned to similar orientations (Wenderoth and Johnstone, 1988), and as such, should be reduced in magnitude if cortical suppression is deficient in SZ. It is worth noting that although levels of orientation SS in SZ were statistically indistinguishable from controls’, there was a trend in the same direction as for contrast and size: biases were lower in the patient group, raising the issue of statistical power. With a greater sample size it is possible that a group difference may have been uncovered. The findings of Yang et al. (2013) make this unlikely however, as they report *elevated* biases in their patient group relative to controls, although this trend did not reach significance. In addition, as noted earlier, Yang et al. (2013) used unlimited exposure times, which may be critical to their findings.

There are several possible explanations as to why orientation SS is relatively normal in SZ. First, orientation SS may rely on distinct mechanisms and cortical networks from other forms of SS that have been implicated in SZ (e.g. contrast SS), and these may be relatively spared in SZ. This also seems unlikely however, as the suppressive inputs that drive contrast SS itself (which is affected in SZ) are tuned for orientation (Chubb et al., 1989; Solomon et al., 1993). An alternative possibility is that the stimuli used here (and previously) were suboptimal for capturing a deficit in the patient group. We used relatively broad-band reference and surround textures with mean orientations separated by 20°. This design may have driven broadly tuned mechanisms of suppression, which are seemingly unaffected in SZ. Thus, there is evidence that orientation tuning curves are abnormally broad in SZ (Rokem et al., 2011), and further, that suppression deficits are specific to closely oriented (i.e. near-parallel) stimuli. In a study of contrast SS using oriented narrow-band stimuli suppression from a parallel surround was found to be reduced in SZ (relative to controls), whilst suppression from an orthogonal surround was, if anything, elevated (see Yoon et al., 2009, Figure 2B). Similarly, in a recent study of contour detection in SZ, whilst the presence of near-parallel flankers selectively impaired performance in the patient group relative to controls (putatively via elevated suppression of activity driven by the contour), near-orthogonal flankers had no such differential effect on the two groups (Robol et al., in press). Therefore, in future studies it may be more informative to test for orientation effects using closely oriented narrow-band reference and surround textures.

With respect to the underlying pathophysiology of visual dysfunction in SZ there are a number of candidate neurotransmitter systems that have been heavily implicated in the disorder, most notably GABA (Wassef et al., 2003), dopamine (Howes and Kapur, 2009) and glutamate (Javitt, 2010). However, the data we report are largely consistent with studies that highlight the importance of reduced levels of GABA in SZ. GABA has been linked to reduced SS (Yoon et al., 2010) and broader orientation tuning (Rokem et al., 2011) in SZ, and GABA-mediated inhibition is thought to be critical to a number of other tasks that are affected in SZ, e.g. orientation discrimination (Edden et al., 2009; Robol et al., in press) and contour integration (Silverstein et al., 2000, 2006; Uhlhaas et al., 2006a,b). Further, a number of studies have highlighted the potential benefits of targeting GABAergic networks with pharmacological interventions (Wassef et al., 1999). For example, improvements in cognitive function have been demonstrated in patients with chronic SZ following administration of a GABA type A receptor sub-unit selective agonist (Lewis et al., 2008), whilst pharmacological induction of a GABA deficit in patients with SZ has been shown to exacerbate psychotic symptoms and perceptual abnormalities (Ahn et al., 2011). However, a meta-analysis of randomized controlled drug studies comparing the use of benzodiazepines, which directly enhance the effects of GABA at the receptor level, to anti-psychotics or placebo concluded that current evidence does not warrant their use in the treatment of SZ (Volz et al., 2007), although the authors simultaneously emphasized the poor quality and relative paucity of existing studies. Consequently, further research is needed to determine whether abnormalities of GABAergic function could underlie the visual dysfunction we report here.

To investigate a possible relationship between anti-psychotic medication and surround suppression in the patient group, we tested for correlations between patients’ behavioral measures and prescribed drug dosages following conversion into chlorpromazine equivalents; no significant correlations were found. However, this approach does not provide a particularly rigorous test of medication-related confounds: equivalent doses are calculated on the basis of DA receptor type-2 binding affinity only, whilst anti-psychotics typically affect multiple neurotransmitter systems and receptor types. Nonetheless, several other lines of evidence lead us to believe that the effects we report are unlikely to be driven by medication. First, the main effect reported (reduced SS) has been observed in disparate samples of patients with a wide range of medication regimes (Dakin et al., 2005; Tadin et al., 2006; Uhlhaas et al., 2006a,b; Yoon et al., 2009, 2010; Barch et al., 2012; Yang et al., 2013). Further, we have previously shown that the effect (for contrast) was specific to patients with SZ, and did not extend to a clinical control group with bipolar disorder, several of whom were also treated with low-dose anti-psychotics (Dakin et al., 2005). Lastly, the effects we report were not seen in all dimensions tested.

Other potential confounds of a non-visual nature, e.g. differences in general cognitive function, attention, or motivation (see O’Carroll, 2000; Rund, 1998 for reviews), should also be considered. Barch et al. (2012), using a similar contrast SS paradigm, have recently shown that following exclusion of participants on the basis of high lapse rates – a putative measure of attentional engagement – group differences in levels of SS between patients and controls essentially disappeared. The authors suggest that their findings imply reduced contrast SS in SZ may be largely due to impaired mechanisms of attention. The underlying logic is that the patients failed to attend to the stimulus on a significant proportion of trials, thereby leading to random responses that masked inherent (perceptual) biases. However, the effect size they report prior to filtering is relatively small (Cohen’s *d* = 0.27–0.31). An alternative interpretation of the data therefore, is that by filtering outliers the authors simply reduced their power, and hence, chance of finding a significant effect. In support of this possibility it is worth noting that even after filtering out 25% of their patient data (66 out of 262), their reported effect remained near-significant (*P* = 0.08 – ANOVA interaction), and in fact, *would be* significant if a single-tailed test were applied, an approach that would be justified on the basis of previous published data. Hence, it may be too premature to conclude that reduced SS in SZ is due to impaired mechanisms of attention.

With respect to our own data, there are a number of reasons why we believe that the main findings reported, i.e. reduced contrast and size SS in the patient group, are unlikely to be due to an inability to attend to the task at hand. First, if this were the case, we would expect to find consistent inter-group differences across all tasks. Thus, why would lapses of attention be specific to a subset of visual judgments? Even if one *were* to posit a possible mechanism by which effects might be specific to a subset of tasks, for example if they were differentially demanding of available attentional resources, this leads to a clear prediction: that inter-group differences in the size of associated parameter CIs should be maximal – or at least evident – on those very tasks which are seemingly affected in SZ (i.e. judgments of contrast and size). In fact, the findings we report show clearly that this predicted relationship is *not* upheld: CIs were larger in the patient group for the orientation task only (for which biases did *not* differ between groups), and did *not* differ for the contrast task (on which biases *did* differ between groups). In addition, there was no correlation between biases and thresholds on individual tasks as one might also expect if levels of bias were an artifact of attentional lapses.

Taken together, the findings reported are inconsistent with inter-group differences in levels of SS being driven by higher attentional lapses in our patient group. This conclusion is further reinforced by the fact that the effect we report is evident following removal of extreme outliers defined on the basis of CIs, and the inverse weighting of individual data-points according to CIs. Both of these data processing stages would have excluded – and minimized the contribution of – participants who were unable to attend to the task, thereby fulfilling a similar function to excluding participants on the basis of lapse rates. Nonetheless, the inclusion of catch-trials to the basic experimental design represents an invaluable improvement; however, we would recommmend integrating these trials into the data-fitting stage by including a lapse rate as an additional parameter to the psychometric function. In this way data need not be discarded and power is retained.

Although we have argued against an explanation of our findings based on the notion of increased attentional lapses in the patient group, is it possible that some other inter-group difference in patterns of attentional deployment could be invoked? One possibility that should be considered is that patients with SZ simply have a smaller spotlight of attention than control participants. If this were the case, then patients might attend less to the high-signal surround, thereby attenuating its suppressive effects (Shulman, 1992; Sundberg et al., 2009). In support of this possibility, there is evidence that on any given fixation, patients with SZ process information from a smaller area of the visual field than do controls (Elahipanah et al., 2011). In addition, patients with SZ are less sensitive at detecting peripheral stimuli during a concurrent foveal discrimination task (Cegalis and Deptula, 1981) or a visual search task (Elahipanah et al., 2010). However, as with attentional lapses, any explanation of this kind needs to address the fact that reported group differences are specific to a subset of SS effects (i.e. luminance judgments are not affected). Further, it is worth noting that for contrast judgments at least (the most robust finding to date), reduced SS in the patients persists when stimuli are maintained onscreen until a response is given (Yang et al., 2013), conditions under which observers are likely to have made multiple saccades, thereby minimizing any influence of differences in the spotlight of attention.

In conclusion, our data suggest that reduced SS characterizes the visual system in SZ, affecting visual judgments of relative contrast and size. However, as not all visual dimensions were affected, we must reject the hypothesis that attenuated suppression is a general (ubiquitous) property of the brain in SZ. Specifically, as the data did not implicate abnormal *luminance* gain control – a finding that has been reported previously – we propose that the putative dysfunction may be predominantly cortical in origin. Considered within the context of previous research, our data suggest that an attenuated contrast SS effect represents a robust and pronounced feature of SZ, with clinical/diagnostic value. On the other hand, whilst the weight of evidence points toward the existence of analogous abnormalities in the contextual processing of other visual dimensions (particularly size), several effects (e.g. for orientation and motion) seem to be more fragile. Future studies involving parametric manipulation of stimulus parameters and testing conditions are therefore needed if critical variables are to be identified and remaining discrepancies in the literature are to be resolved.

## Conflict of Interest Statement

The authors declare that the research was conducted in the absence of any commercial or financial relationships that could be construed as a potential conflict of interest.
