# Corrigendum: Visual surround suppression in schizophrenia

**DOI:** 10.3389/fpsyg.2019.01898

**Published:** 2019-08-22

**Authors:** Marc S. Tibber, Elaine J. Anderson, Tracy Bobin, Elena Antonova, Alice Seabright, Bernice Wright, Patricia Carlin, Sukhwinder S. Shergill, Steven C. Dakin

**Affiliations:** ^1^Institute of Ophthalmology, University College London, London, UK; ^2^NIHR Biomedical Research Centre at Moorfields Eye Hospital, London, UK; ^3^Institute of Cognitive Neuroscience, University College London, London, UK; ^4^Institute of Psychiatry, King's College London, London, UK; ^5^Department of Cognitive, Perceptual and Brain Sciences, University College London, London, UK

**Keywords:** schizophrenia, surround suppression, cortex, contrast, luminance, size, orientation, perception

In the original article, we neglected to include the funder “European Research Council (ERC), ERC Consolidator Award” to Professor Sukhwinder S. Shergill.

A correction has therefore been made to **Acknowledgments** and the correct statement appears below:

“This work was funded by the Wellcome Trust. SS received funding from the European Research Council (ERC) Consolidator Award. The authors gratefully acknowledge the generous assistance of Cambian Healthcare in the data collection. The authors have no financial interests to disclose.”

The authors apologize for this error and state that this does not change the scientific conclusions of the article in any way. The original article has been updated.

